# Evaluation of UroVysion for Urachal Carcinoma Detection

**DOI:** 10.3389/fmed.2020.00437

**Published:** 2020-08-27

**Authors:** Zhiquan Hu, Chunjin Ke, Zheng Liu, Xing Zeng, Song Li, Hua Xu, Chunguang Yang

**Affiliations:** Department of Urology, Tongji Hospital Affiliated to Tongji Medical College of Huazhong University of Science and Technology (HUST), Wuhan, China

**Keywords:** urachal carcinoma, urothelial carcinoma, fluorescence *in situ* hybridization, UroVysion, differential diagnosis

## Abstract

**Background:** Patients with hematuria who are positive for urinary fluorescence *in situ* hybridization (FISH) are generally considered to have urothelial carcinoma. We determined whether UroVysion FISH could be used for the diagnosis of urachal carcinoma.

**Methods:** Seven cases of urachal carcinoma with haematuria subjected to FISH analysis were retrospectively analyzed in our hospital from May 2012 to November 2019. Paraffin-embedded tissue sections from one FISH-positive and one FISH-negative urachal carcinoma were processed in strict accordance with the instructions of the UroVysion kit. Meanwhile, FISH data from the other 414 hematuria patients were collected as controls.

**Results:** All 7 patients with urachal carcinoma were diagnosed with adenocarcinoma. According to Sheldon stage, six patients had stage IIIa and one patient had stage IVb. The sensitivity and specificity of urinary FISH for the diagnosis of urachal carcinoma were 71.43% (5/7) and 94.61% (281/297), respectively. The rates of polysomy for chromosomes 3 and 7 in positive patients were both 100% (5/5), whereas the rate of polysomy for chromosome 17 was 40% (2/5), and the chromosome 9p21 region (p16) gene deletion rate was 20% (1/5). Histological assessment and cytological FISH were consistent for urachal carcinoma. No significant difference was observed in the diagnostic efficacy between urachal carcinoma and urothelial carcinoma (71.43 vs. 87.18%, *P* = 0.245).

**Conclusions:** Taken together, UroVysion FISH was found to be positive in a high proportion of pathologically confirmed urachal carcinoma of late stage with hematuria. Its chromosomal aberrations may be different from those of urothelial carcinoma, but more studies are needed to clarify their genetic background. Not all tumors showing abnormalities by FISH are urothelial carcinomas.

## Introduction

The urachus is a tubular structure extending from the top of the bladder to the umbilicus during embryonic development. Before birth and in infancy, the tubular structure disappears and degenerates into the median umbilical ligament, which is located in the median umbilical fold (1). Urachal carcinoma is a rare genitourinary tumor originating from the urachal tract, with an annual incidence of ~1/5,000,000 (2). This cancer accounts for 0.35 to 0.7% of all bladder cancers (3, 4). Although the incidence of the disease is low, it is difficult to effectively control the tumor by surgical resection or systemic treatment, and patient safety is seriously threatened. Therefore, preoperative diagnosis is particularly important.

UroVysion fluorescence *in situ* hybridization (FISH) is a sensitive and specific method for the diagnosis of urothelial carcinoma. The fluorescently labeled DNA probe is denatured into a single strand, after which it hybridizes with the denatured chromosome or nuclear target DNA. The DNA is then observed under a fluorescence microscope. This method has been approved for the screening of patients with hematuria and the monitoring of recurrent urothelial carcinoma. Moreover, many studies have been performed using the application of FISH in urothelial carcinoma (5–10). Thus, patients with hematuria who are positive for urinary FISH are often diagnosed with urothelial carcinoma.

Kipp et al. (11) retrospectively assessed non-urothelial carcinoma (including urachal carcinoma) for chromosomal abnormalities in paraffin tissue sections by FISH with the UroVysion probe set and found that chromosomal abnormalities in urothelial carcinoma are also common in rarer histological variants of bladder cancer. Yang et al. (12) investigated the value of FISH in bladder paraganglioma using urine specimens. In these studies, diagnostic efficacy of UroVysion for rarer histological cancer was not demonstrated. Moreover, the results were not compared between paraffin tissue sections or urine cytology specimens. Our previous clinical work showed that urinary FISH can also show positive signs in urachal carcinoma, which motivated this research. Therefore, this study focused on the diagnostic value of FISH in patients with urachal carcinoma and compared the consistency of histological and cytological FISH results.

## Patients and Methods

### Patients and Samples

After the approval of the Department Review Committee, seven cases of urachal carcinoma with urinary FISH analysis data were collected from the medical records department of our hospital from May 2012 to November 2019 (see Table 1 for general clinical information) and were included in this study. The inclusion criteria were as follows: (1) patients with urachal carcinoma confirmed by pathology and clinical data; (2) patients underwent a urinary FISH test before surgery; and (3) systemic treatment was administered after surgery. The exclusion criteria were as follows: patients with a history of malignant tumors or urinary tract infections. At the same time, data on the other 414 patients who underwent FISH testing because of hematuria in our hospital were collected, and these patients served as the control group. Among them, 117 cases were diagnosed with urothelial carcinoma, and 297 cases were diagnosed with non-urothelial tumors. In addition, the paraffin sections of the aforementioned cases A and F were submitted to the pathology department for histological FISH analysis.

**Table 1:** General clinical information of 7 patients with urachal carcinoma.

**Patients**	**Sex**	**Age (years)**	**First symptom**	**FISH (+/–)**	**Stages**	**Treatments**	**Pathology**
A	Male	25	Painless gross hematuria	+	IIIa	EPC + chemo + radio + targeted	Medium-differentiated adenocarcinoma
B	Male	54	Umbilical blood, purulent discharge, hematuria	+	IVb	Chemo	Poorly differentiated adenocarcinoma
C	Male	30	Painless intermittent gross hematuria	+	IIIa	EPC + chemo + radio	Mucous adenocarcinoma
D	Male	46	Gross hematuria	+	IIIa	RC + RBS	Medium/poor-differentiated adenocarcinoma
E	Male	68	Painless gross hematuria	+	IIIa	EPC	Urachal adenocarcinoma
F	Female	49	Painless gross hematuria	**–**	IIIa	RC + IBS + PLND	Medium-differentiated adenocarcinoma
G	Female	50	Gross hematuria	**–**	IIIa	PC + chemo	Medium-differentiated adenocarcinoma

*RC, radical cystectomy; IBS, ileal bladder replacement; RBS, rectal bladder replacement; EPC, extended partial cystectomy; PC, partial cystectomy; Chemo, chemotherapy; Radio, radiotherapy; PLND, pelvic lymph node dissection*.

### FISH Processing

The FISH DNA probe was provided by Beijing Jinpujia Medical Technology Co., Ltd., and consists of two combinations of Chromosome Region-Specific Probe 3[CSP3 (green)]/CSP7 (red) and gene locus–specific probe p16[GLPp16 (red)]/CSP17 (green). The main reagents needed in the experimental operation are prepared and used in strict accordance with the instructions of FISH, which are mainly provided by Beijing Jinpu Jia Medical Technology Co., Ltd., and the supporting reagents are provided by our hospital reagent room supply (Supplementary Material for details). First, the 5-μm paraffin-embedded sections were placed in a 56°C oven overnight and were then placed in xylene for multiple deparaffinization steps. The slides were dehydrated in 100% ethanol for 5 min at room temperature, and after air drying for 3 min, the slides were immersed in 10 mmol/L citric acid buffer (80°C; pH 6.0) for 45 min, followed by immersion in 2 × saline-sodium citrate (SSC) for 5 min at 37°C. The samples were then digested in 0.2% pepsin solution (2,500–3,500 U/mg) for 48 min at 37 °C, after which the sections were placed in 70, 85, and 100% ethanol solutions. Each section was dehydrated in ethanol for 2 min. Next, 10 μL of the UroVysion probe (Beijing Jinpujia Medical Technology Co., Ltd) was applied to the target tissue area. The probe and target DNA were denatured and hybridized on a HYBrite instrument at 80°C for 3 min and hybridized at 37°C for 16 to 18 h. After hybridization, any unbound probe was removed by washing in 2 × SSC/0.1% NP-40 at 76°C for 2 min, followed by 1 min at room temperature in 2× SSC/0.1% NP-40. Then, 10 μL of DAPI II counterstain was applied, and the slides were cover-slipped. Fluorescence *in situ* hybridization results were analyzed by two professional certified double-blind pathologists with 10 years of work experience. A minimum of 25 tumor cells were visualized and evaluated for these chromosomal changes. If no abnormalities were detected, then the remaining cells were counted until a sufficient number of cells with chromosomal abnormalities were found or until 200 cells were evaluated. A positive result was the presence of ≥4 (or >10%) cells with gains of 2 or more of chromosomes 3, 7, and 17. In the case of chromosome nine, a positive result was one in which more than 12 cells showed zero 9p21 signals (13).

### Statistical Analysis

SPSS 23.0 was used for the statistical analysis. The statistical data were analyzed with the independent *t*-test and χ^2^-test. *P* < 0.05 was considered statistically significant.

## Results

### Application of Urinary FISH for the Detection of Urachal Carcinoma and Urothelial Cancer

Overall, data on 31 patients with urachal carcinoma were collected from the medical records department, and of them, seven patients met the inclusion and exclusion criteria. According to the Sheldon stage, six of the remaining seven patients had stage IIIa, and one had stage IVb, and the most common symptom was hematuria. The urinary FISH assay was positive in five cases and negative in two cases. The sensitivity and specificity for the diagnosis of urachal cancer were 71.43% (5/7) and 94.61% (281/297), respectively. The control group contained 414 cases, and the urothelial carcinoma group contained 117 cases; 102 and 15 cases of which were positive and negative, respectively. The non-urothelial tumor group included 297 cases, 16 and 281 of which were positive and negative, respectively. It can be seen that the sensitivity and specificity of urinary FISH for the diagnosis of urothelial carcinoma are 87.18% (102/117) and 94.61% (281/297), respectively. No significant difference was observed in the diagnostic efficacy between urachal carcinoma and urothelial carcinoma (71.43 vs. 87.18%, *P* = 0.245) (Table 2).

**Table 2:** Comparison of positive detection rates of urinary FISH in the urachal carcinoma group, urothelial cancer group, and non-urothelial tumor group [*n* (%)].

**Groups**	**FISH**		***P***
	**+**	**–**	
Urachal carcinoma group (*n* = 7)	5 (71.43)	2 (28.56)	*P* = 0.245
Urothelial carcinoma group (*n* = 117)	102 (87.18)	15 (12.80)	
Non-urothelial carcinoma group (*n* = 297)	16 (5.40)	281 (94.60)	^a^*P* < 0.0001, ^b^*P* < 0.0001

*P = 0.245 refers to the comparison between the urachal carcinoma group and the urothelial carcinoma group*.

a *P < 0.0001 refers to the comparison between the urachal carcinoma group and the non-urothelial tumor group. The Fisher χ^2^-test was used. To compare the urothelial carcinoma group with the non-urothelial tumor group, Pearson χ^2^-test was used, χ^2^ = 275.55*.

b *P < 0.0001*.

### Chromosome Aberrations in Urinary FISH-Positive Urachal Carcinoma Patients

**Figure 1:**
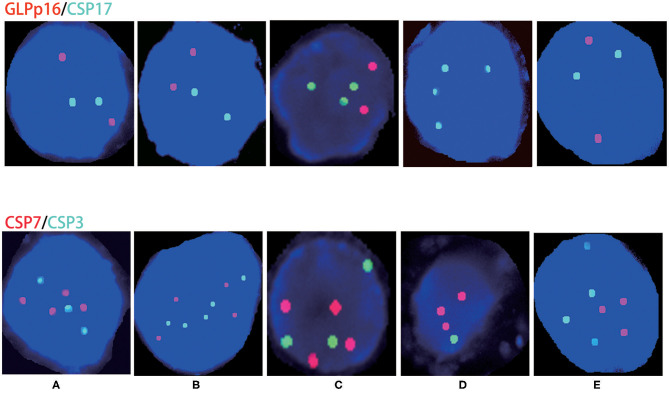
Chromosome aberrations in patients with urinary FISH-positive urachal carcinoma. **(A–E)** Correspond to cases **(A–E)**; red represents CSP7 and GLPp16, whereas green represents CSP7 and CSP17.

The five positive patients were male: four were stage IIIa, and one was stage IVb. The aberration rate of chromosomes 3 and 7 was 100% (5/5), the aberration rate for chromosome 17 was 40% (2/5), and the chromosome 9p21 region (p16) gene deletion rate was 20% (1/5). Specific abnormal conditions are shown in Table 3 and Figure 1.

**Table 3:** Chromosome aberrations in patients with FISH-positive urachal carcinoma.

**Cases sex stages**			**Chromosome polysomy/gene deletion**			
			**3#**	**7#**	**17#**	**GLPp16**
A	Male	IIIa	1	1	0	0
B	Male	IVb	1	1	0	0
C	Male	IIIa	1	1	1	0
D	Male	IIIa	1	1	1	1
E	Male	IIIa	1	1	0	0

*1, indicates chromosome polysomy or gene deletion; 0, indicates none*.

### Comparison of Histological and Cytological FISH Analysis Results

**Figure 2:**
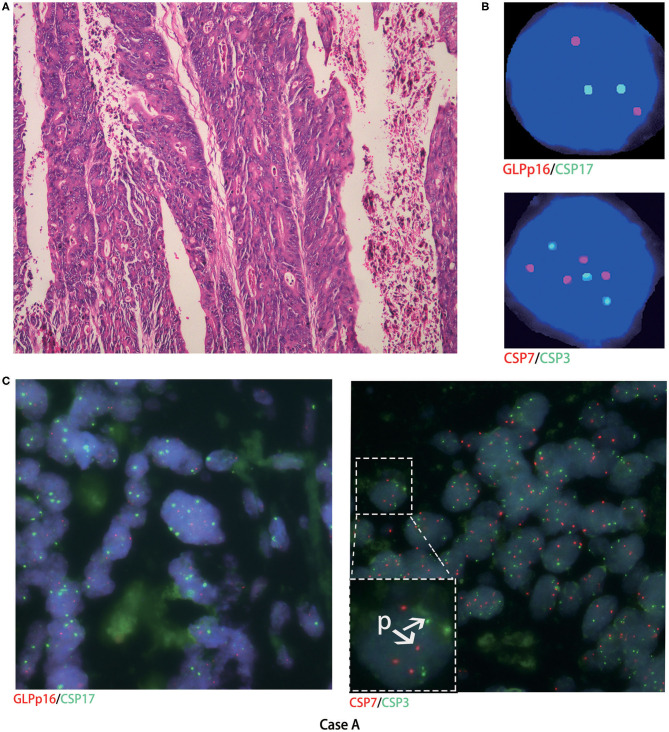
Cytological and histological chromosome aberrations in case A. **(A)** Microscopy revealed moderately differentiated adenocarcinoma (hematoxylin-eosin staining, magnification ×200); **(B)** case A urinary FISH showed chromosomes 3 and 7 polysomy, no chromosome 17 polysomy, and no chromosome 9p21 region gene deletion; **(C)** histological FISH in case A also showed chromosomes 3 and 7 polysomy (as shown in P), no chromosome 17 polysomy, and no chromosome 9p21 region gene deletion; red represents CSP7 and GLPp16, and green represents CSP7 and CSP17.

**Figure 3:**
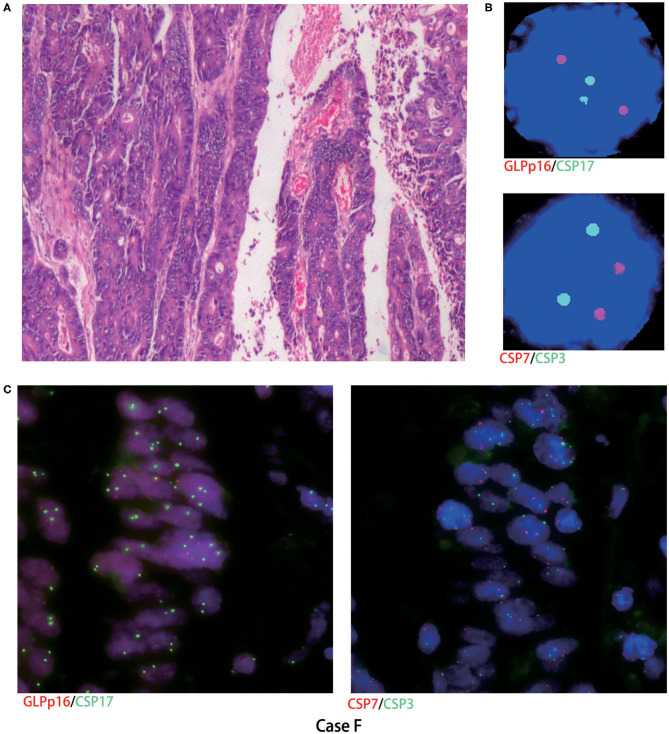
Cytological and histological FISH test results in case F. **(A)** Microscopy revealed moderately differentiated adenocarcinoma (hematoxylin-eosin staining, magnification ×200); **(B,C)** case F urinary and histological FISH testing was negative; red represents CSP7 and GLPp16, and green represents CSP7 and CSP17.

Histological FISH performed in case A showed chromosomes 3 and 7 polysomy, no chromosome 17 polysomy, and no chromosome 9p21 region (p16) gene deletion (Figure 2). Histological FISH testing in case F was negative (Figure 3), consistent with cytological FISH results. These findings might indicate that the tumor cells shed into urine originated from tumor tissue.

## Discussion

Although the incidence of urachal carcinoma is very low, it is highly malignant. Patients are usually at a later stage at diagnosis, whereas early urachal carcinoma is often confined to the umbilical duct without obvious clinical symptoms. Clinical symptoms mostly appear when the tumor invades or breaks through the bladder. Hematuria, as the most common symptom of urachal carcinoma (14), is also the most common symptom of bladder tumors, ~90% of which are urothelial carcinomas. UroVysion FISH was originally used to diagnose urothelial carcinoma rather than non-urothelial carcinoma. We found that FISH could also yield positive results in urachal carcinoma in previous clinical practice, which motivated this research.

Numerous studies have evaluated the efficacy of FISH in urothelial carcinoma (5–10), whereas data on the evaluation of UroVysion probes in non-urothelial carcinoma are scarce. This study found that the positive rate of FISH in urachal carcinoma of late stage with hematuria was rather high at 71.43%, which was not significantly different from that in urothelial carcinoma (71.43 vs. 87.18%, *P* = 0.245). At the same time, our study showed that the polysomy rate of chromosomes 3 and 7 was 71.43% (5/7), and that of chromosome 17 was 28.57% (2/7). The chromosome 9p21 region (p16) gene deletion rate was only 14.29% (1/7). However, in a multicenter big data study conducted by Zhou et al. (10) in China, the polysomy of chromosomes 3, 7, and 17 and gene changes at 9p21 region (p16) accounted for 71.3% (2941/4125), 72.2% (2978/4125), 67.4% (2780/4125), and 72.9% (3007/4125) of urothelial carcinoma cases, respectively. Therefore, the chromosome 9p21 region (p16) gene deletion in urachal carcinoma seems to occur less frequently than in urothelial carcinoma (16.67 vs. 72.9%, *P* = 0.008).

To compare chromosomal aberrations in urine exfoliative cytological FISH and histological FISH, we performed histological FISH of urachal carcinoma, which was not reported previously. Histological FISH in case A also showed chromosomes 3 and 7 polysomy, no chromosome 17 polysomy, and no chromosome 9p21 gene deletion. Histological FISH in case F was negative. Thus, it might indicate that the tumor cells shed in urine originated from urachal carcinoma.

Studies have shown that for patients with invasive and highly malignant tumors, the tumor cells have many genetic abnormalities (15, 16). The principle of FISH is to use fluorescently labeled GLPp16 site-specific probes and CSP3/CSP7/CSP17 chromosomal centromere-specific probes that are hybridized *in situ* with target DNA. If the tumor cells have aberrations in chromosomes 3, 7, 17, or 9p21, and the cancerous cells are shed in sufficient quantities into the urine, urinary FISH may be positive in theory. This study also justifies this approach. Kipp et al. (11) and Reid-Nicholson et al. (13) found that metastatic colon cancer, cervical cancer that metastasizes to the bladder, and primary squamous cell carcinoma and small cell carcinoma of the bladder exhibit FISH-positive histological FISH results, which is consistent with our speculation. These results indicate that FISH positivity should not be simply interpreted as urothelial carcinoma. Additionally, whether the probe can be more scientifically modified to increase its sensitivity and specificity for urachal carcinoma remains to be determined.

Of course, our study has some limitations. Because the incidence of urachal carcinoma is very low, the number of cases is small, and the conclusions should be better verified in more patients. Moreover, studies (17–19) have shown that urinary FISH can be used to predict the recurrence of urothelial carcinoma. Therefore, in theory, FISH may also be useful for urachal carcinoma monitoring after surgery, which requires further research.

## Conclusion

In summary, UroVysion FISH was found to be positive in a high proportion of urachal carcinoma of late stage, which is a potential diagnostic indicator of urachal carcinoma. Its chromosomal aberrations may be also different from those of urothelial carcinoma, which may aid in their differential diagnosis. Fluorescence *in situ* hybridization positivity should not be simply interpreted as urothelial carcinoma.

### Clinical Practice Points

- UroVysion FISH is a potential diagnostic indicator of late-stage urachal carcinoma with hematuria.
- Fluorescence *in situ* hybridization can be positive in a number of different diseases presenting with hematuria.
- Chromosomal aberrations of urachal carcinoma seem to be different from those of urothelial carcinoma, which may aid in their differential diagnosis.

## Data Availability Statement

The raw data supporting the conclusions of this article will be made available by the authors, without undue reservation.

## Ethics Statement

The studies involving human participants were reviewed and approved by Medical Ethical Committee of Tongji Hospital of Huazhong University of Science and Technology. Written informed consent for participation was not required for this study in accordance with the national legislation and the institutional requirements.

## Author Contributions

ZH: project administration and resources. CK: writing—original draft. ZL: data curation and formal analysis. XZ: software and supervision. SL: writing—review and editing. HX: review and editing. CY: conceptualization, funding acquisition, and writing—review and editing. All authors contributed to the article and approved the submitted version.

## Conflict of Interest

The authors declare that the research was conducted in the absence of any commercial or financial relationships that could be construed as a potential conflict of interest.
